# Acute and chronic inflammation alter immunometabolism in a cutaneous delayed-type hypersensitivity reaction (DTHR) mouse model

**DOI:** 10.1038/s42003-022-04179-x

**Published:** 2022-11-15

**Authors:** Laimdota Zizmare, Roman Mehling, Irene Gonzalez-Menendez, Caterina Lonati, Leticia Quintanilla-Martinez, Bernd J. Pichler, Manfred Kneilling, Christoph Trautwein

**Affiliations:** 1 Werner Siemens Imaging Center, Department of Preclinical Imaging and Radiopharmacy, Eberhard Karls University of Tübingen, Röntgenweg 13, 72076 Tübingen, Germany; 2 Cluster of Excellence iFIT (EXC 2180) “Image-Guided and Functionally Instructed Tumor Therapies”, Eberhard Karls University of Tübingen, Röntgenweg 11, 72076 Tübingen, Germany; 3 Institute of Pathology and Neuropathology, Comprehensive Cancer Center, Eberhard Karls University of Tübingen, Liebermasterstraße 8, 72076 Tübingen, Germany; 4 Center for Preclinical Research, Fondazione IRCCS Ca’ Granda Ospedale Maggiore Policlinico, Via Pace 9, 20100 Milan, Italy; 5 Department of Dermatology, Eberhard Karls University of Tübingen, Liebermeisterstraße 25, 72076 Tübingen, Germany

**Keywords:** Metabolomics, Chronic inflammation, Autoimmune diseases, Metabolic pathways

## Abstract

T-cell-driven immune responses are responsible for several autoimmune disorders, such as psoriasis vulgaris and rheumatoid arthritis. Identification of metabolic signatures in inflamed tissues is needed to facilitate novel and individualised therapeutic developments. Here we show the temporal metabolic dynamics of T-cell-driven inflammation characterised by nuclear magnetic resonance spectroscopy-based metabolomics, histopathology and immunohistochemistry in acute and chronic cutaneous delayed-type hypersensitivity reaction (DTHR). During acute DTHR, an increase in glutathione and glutathione disulfide is consistent with the ear swelling response and degree of neutrophilic infiltration, while taurine and ascorbate dominate the chronic phase, suggesting a switch in redox metabolism. Lowered amino acids, an increase in cell membrane repair-related metabolites and infiltration of T cells and macrophages further characterise chronic DTHR. Acute and chronic cutaneous DTHR can be distinguished by characteristic metabolic patterns associated with individual inflammatory pathways providing knowledge that will aid target discovery of specialised therapeutics.

## Abstract (web summary)

Nuclear magnetic resonance spectroscopy-based tissue metabolomics is used to define detailed temporal signatures of acute and chronic inflammation in cutaneous delayed-type hypersensitivity reaction.

## Introduction

Immunometabolism is crucial to fine-tune and modulate inflammatory processes and thus can terminate or promote inflammation directed to the final pathophysiological phenotype^1^. Several autoimmune conditions, such as rheumatoid arthritis or psoriasis vulgaris, are characterised as T-cell-driven inflammatory immune responses^2,3^. Dynamically progressing chronic inflammatory autoimmune processes are also frequently associated with a loss of function, scar formation and permanent tissue damage, such as cartilage and joint destruction.

Inflammation is highly interconnected with reactive oxygen and nitrogen species (RONS) production^4^. RONS are necessary for the appropriate inflammatory response and immune cell signalling. Alternatively, excessive RONS production is extremely toxic not only to pathogens but also to endogenous cells, resulting in increased synthesis of RONS-scavenging metabolites such as ascorbate and glutathione. Triggering inflammation results in metabolic reprogramming, which leads to temporary metabolic changes; some pathways, such as lipid metabolism^5^ and the tricarboxylic acid (TCA) cycle^6^, are disrupted due to the increasing need for RONS scavengers and energy production^7^. If the inflammatory state is prolonged, it can eventually lead to permanent site-specific and systemic dysregulation and tissue damage. Inflammation progression and cellular homoeostasis can thus be characterised by metabolite changes, offering a potential wider understanding and points for early intervention^8^.

Recently, we noninvasively characterised the temporal dynamics of RONS^9^ in vivo and the differential impact of different RONS sources^8^ in our T-cell-driven trinitrochlorobenzene (TNCB)-induced acute and chronic contact delayed-type hypersensitivity reaction (DTHR) prototypic preclinical model^10–13^, which aids in determining the underlying mechanisms of T-cell-driven autoimmune diseases.

Nuclear magnetic resonance (NMR) spectroscopy and mass spectrometry-based metabolomics have been previously broadly applied to characterise inflammation in various models, such as murine tissue^14,15^, patient blood^16,17^, urine^18^, and biopsy tissue^19^, indicating a crucial metabolomics role in inflammation characterisation during disease progression^20,21^.

We employed the established prototypic cutaneous DTHR model^13^, mediated by IFN-γ-producing CD4^+^ (Th1 cells) and CD8^+^ T cells (Tc1 cells)^13,22,23^, to characterise the temporal dynamics of the metabolic signature during acute and progressive inflammation and to uncover the underlying mechanisms of T-cell-driven inflammatory diseases.

The focus of this study was to identify the metabolic alterations, pathways and regulators that indicate the switch from acute to chronic inflammation and the related biomolecular events occurring during progressive T-cell-driven inflammation. Hereby it was found that acute and chronic DTHR yielded significantly different metabolite concentration profiles related to tricarboxylic acid (TCA) cycle activity, energy metabolism, redox metabolism, nucleotide and growth metabolism, and immune cell activity. These findings help to better understand the underlying metabolic events of acute and chronic T-cell-driven inflammatory immune responses and serve as a prerequisite for the development of novel therapeutics.

## Results

### Ear swelling progresses during acute and chronic cutaneous DTHR

**Fig. 1:**
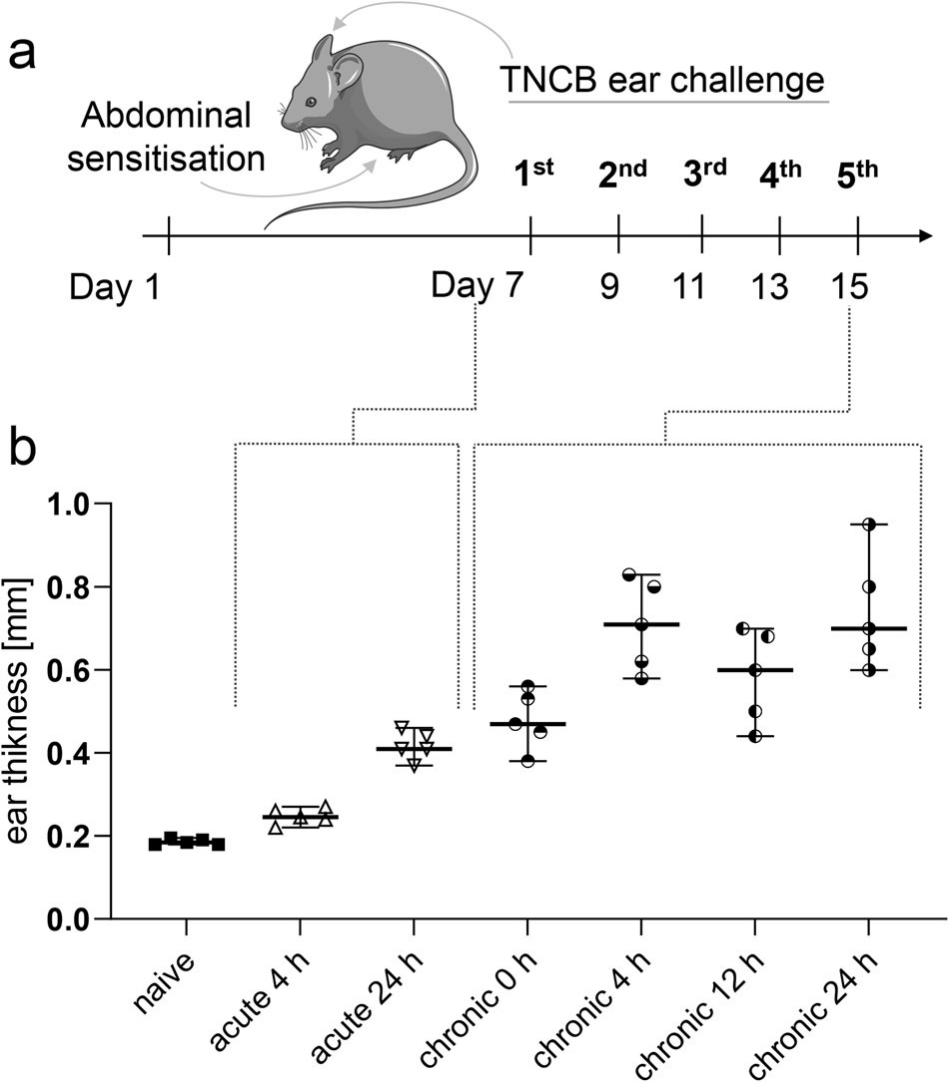
Experimental setup, course of ear swelling response and metabolite concentration profiling. **a** Experimental timeline: Sensitisation with 5% trinitrochlorobenzene (TNCB) at the abdomen on Day 1, followed by TNCB ear challenges (1% TNCB) at the right ear on Day 7 (acute DTHR) and repetitive TNCB ear challenges at Days 9, 11, 13 and 15 (chronic DTHR)). **b** Ear thickness of naive mice and mice with acute (4 h and 24 h after the 1st TNCB challenge) and chronic (0 h – 48 h after the 4th challenge – and 4 h, 12 h and 24 h after the 5^th^ TNCB challenge) cutaneous DTHR (*n* = 4 animals).

Experimental setup of the acute and chronic TNCB-specific cutaneous DTHR model is illustrated in Fig. 1a. The course of the ear swelling response represents a clinical measure of the severity of inflammation (Fig. 1b), characterising the temporal dynamics of the ear swelling response at the point in time of metabolite analysis of the ear tissue of naive mice, mice with acute (1st TNCB ear challenge) and chronic (5th TNCB ear challenge) cutaneous DTHR. During acute cutaneous DTHR, ear thickness significantly increased (*P* = 0.048) from 4 h (0.24 mm +/− 0.02 mm) to 24 h (0.42 mm +/− 0.03 mm) after the 1st TNCB ear challenge, which is the peak of acute inflammation. At 0 h of chronic cutaneous DTHR, 48 h after the 4th TNCB ear challenge, the ear thickness (0.48 mm +/− 0.06 mm) was slightly above the peak of acute cutaneous DTHR. During chronic cutaneous DTHR, the swelling peaked 4 h and 24 h after the 5th TNCB challenge. In fact, ear thickness increased significantly during the course of chronic cutaneous DTHR from 0 h to 4 h (*P* = 0.003) and from 0 h to 24 h (*P* = 0.0007) after the 5th TNCB ear challenge (Fig. 1b).

### Inflamed ears with acute and chronic cutaneous DTHR exhibit a distinct metabolic profile

**Fig. 2:**
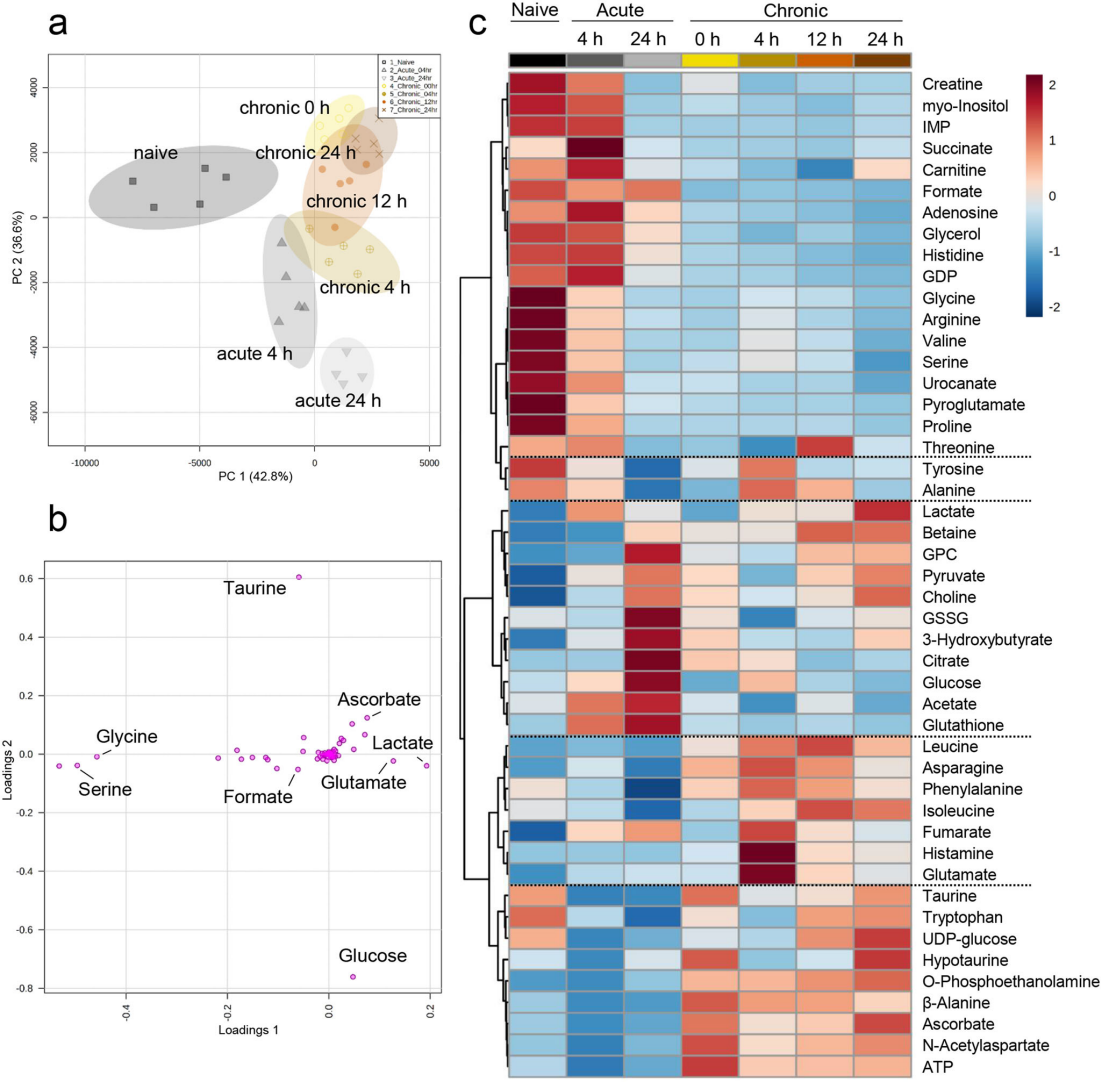
Metabolomics overview of metabolite concentrations in healthy ears and in inflamed ears with acute and chronic cutaneous DTHR. **a** Principal component analysis (PCA) of the first two principal components (PC 1 (42.8%), PC 2 (36.6%)) scores plot illustrating the group separation based on the metabolite concentrations. Naive—dark grey squares, acute 4 h—medium grey upward triangles, acute 24 h—downward light grey triangles, chronic 0 h—yellow empty circles, chronic 4 h—beige dots with cross, chronic 12 h—orange full dots, chronic 24 h—brown exes. **b** PCA loadings plot illustrating the most significant metabolites that drive the principal component separation. **c** Averaged group heatmap illustrating normalised and autoscaled (-2; 2) metabolite concentration changes during acute and chronic cutaneous DTHR, red—relatively high concentration, blue—relatively low concentration. Metabolite list organised by Ward hierarchical clustering using Euclidean distance measure (*n* = 5 animals, except acute 24 h *n* = 4 animals). GDP guanosine diphosphate, IMP inosine monophosphate, GPC sn-glycero-3-phosphocholine, GSSG glutathione disulfide, UMP uracil monophosphate, ATP adenosine triphosphate.

Inflamed ears with acute and chronic cutaneous DTHR were further characterised by their unique metabolite concentration patterns, as shown by the group separation and 95% confidence interval clouds in principal component analysis (PCA) (Fig. 2a). Here, the naive control ears were clearly separated from the inflamed ears with acute and chronic cutaneous DTHR. In addition, PCA also separated inflamed ears with acute DTHR from those with chronic cutaneous DTHR. Interestingly, PCA identified the peaks of acute and chronic cutaneous DTHR (acute, 24 h; chronic, 4 h) in a similar direction in principal component 2. Inflamed ears with chronic cutaneous DTHR, collected 12 h and 24 h after the 5th TNCB ear challenge, exhibited the strongest similarities in metabolite concentration patterns, illustrated by the slight overlap of the 95% confidence interval cloud (Fig. 2a).

When comparing naive ear tissue with acute and chronic DTHR-affected ear tissue in detail, we observed chronic inflammation-dependent specific changes in the metabolic profile, as illustrated in the PCA loadings plot (Fig. 2b) and the heatmap (Fig. 2c and Supplementary Fig. 1). The healthy naive condition was characterised by active amino acid metabolism, nucleotide metabolism and relatively low RONS scavenger concentrations. During the irritative toxic peak of acute cutaneous DTHR, 4 h after the 1st TNCB ear challenge, slight changes towards downregulation of several amino acid concentrations, such as glycine, arginine, valine, serine and proline, were observed. Here, the initial innate immune response was characterised by increased glutathione (GSH), glutathione disulfide (GSSG), acetate, glucose, citrate and fumarate concentrations (Fig. 2b, c). During the peak of the ear swelling response, 24 h after the 1st TNCB ear challenge, we identified increased pyruvate, 3-hydroxybutyrate, choline, sn-glycero-3-phosphocholine (GPC), citrate and glucose concentrations. Meanwhile, amino acids, such as glycine, arginine, valine, serine, proline, isoleucine, and tryptophan, were continuously downregulated during the acute phase of cutaneous DTHR (Fig. 2c).

Chronic cutaneous DTHR was distinctively characterised by increased β-alanine, ascorbate, ATP and taurine concentrations (Fig. 2c and Supplementary Fig. 2). Concentrations of histamine, glutamate, leucine, phenylalanine, alanine and tyrosine in inflamed ears peaked simultaneously with the peak of chronic cutaneous DTHR at 4 h after the 5th TNCB ear challenge, followed by a constant decrease at 12 h and 24 h. During the chronic phase of cutaneous DTHR, glucose, GSH, GSSG and TCA cycle metabolites citrate and succinate remained at low concentrations similar to healthy ears of the naive group (Fig. 2c).

### Histopathological and immunohistochemical characterisation of acute and chronic cutaneous DTHR

**Fig. 3:**
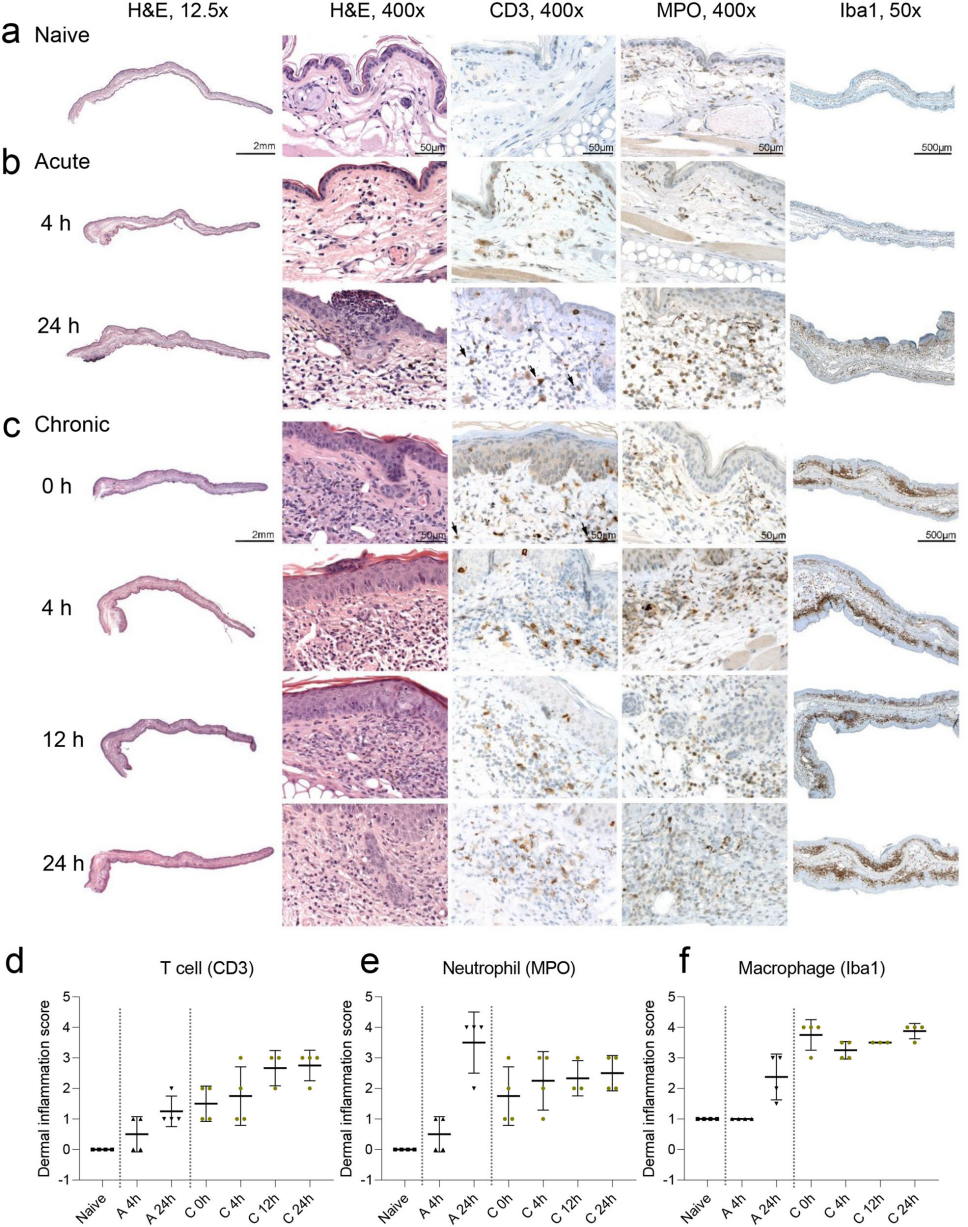
Histopathological (H&E) and immunohistochemical (IHC) characterisation of the inflammatory immune cell infiltrate during acute and chronic cutaneous DTHR. Naive ears (**a**), inflamed ears with acute (**b**) cutaneous DTHR (4 h and 24 h) and (**c**) chronic cutaneous DTHR (0 h, 4 h, 12 h, and 24 h) H&E staining (magnification ×12.5 and ×400, at the scale of 2 mm), CD3, MPO IHC (magnification ×400, at scale of 50 μm), and Iba1 IHC (magnification ×50 and scale of 500 μm). Scoring of dermal CD3 (**d**), MPO (**e**) and Iba1 (**f**) immune cell infiltration presented as a scatter plot: mean +/− SD (0 = no inflammatory infiltrate, 1 = minimal, 2 = mild, 3 = moderate, 4 = severe presence of inflammatory cells, *n* = 4 animals, except chronic 12 h *n* = 3 animals). Grey dashed lines aid visual separation between naive, acute and chronic DTHR.

Histopathological analysis of haematoxylin and eosin (H&E)-stained slices from inflamed ears with acute cutaneous DTHR revealed pronounced oedema with dilated blood vessels and extravasation of PMN, moderate neutrophilic infiltrate, subepidermal neutrophilic abscesses (Munro’s microabscesses) and small crusts at 24 h after the 1st TNCB ear challenge (Fig. 3a, b). Meanwhile, H&E analysis of inflamed ears with chronic cutaneous DTHR showed a pronounced leucocyte infiltrate, hyperkeratosis, acanthosis, dilated blood vessels and an increased number of blood vessels (Fig. 3c).

To further characterise the immune cell infiltrate throughout acute and chronic cutaneous DTHR, we performed immunohistochemistry (IHC), focusing on cells expressing CD3 (T cells), MPO (neutrophils, macrophages) and Iba1 (phagocytes, mainly macrophages). No T cells or neutrophils were present in healthy naive ears, while Iba1 staining revealed the presence of some resident macrophages (Fig. 3a, d–f). During acute cutaneous DTHR, 4 h after the 1st TNCB ear challenge, we observed some MPO- and CD3-positive cells within the vessels but not in the surrounding tissue, indicating an initial infiltration of neutrophils and T cells into the inflamed ears (Fig. 3b, d–f). Notably, 24 h after the 1st challenge, we observed a strongly pronounced infiltration of neutrophils with neutrophil abscesses and crusts in the epidermis, reflecting the characteristics of an early acute inflammatory immune response. In addition, a mild increase in CD3^+^ T cells and macrophages was observed (Fig. 3b, d–f).

At 48 h after the 4th challenge (0 h), the point in time of the 5th TNCB ear challenge, some CD3^+^ T cells together with scarce MPO^+^ cells and a massive accumulation of macrophages in the dermis (Fig. 3c–f) were observed. The infiltration of CD3^+^ T cells and MPO^+^ neutrophils steadily increased from 4 h to 24 h after the 5th TNCB ear challenge, while the presence of macrophages remained at an identical level (Fig. 3c–f).

### Individual metabolites correlate with the severity of the ear swelling response and the specific immune cell subtype infiltration

Our investigations focused on potential correlations between individual metabolite alterations, the degree of ear swelling response, reflecting the severity of inflammation, and the presence of immune cells within the inflamed ears with acute and chronic cutaneous DTHR.

**Fig. 4:**
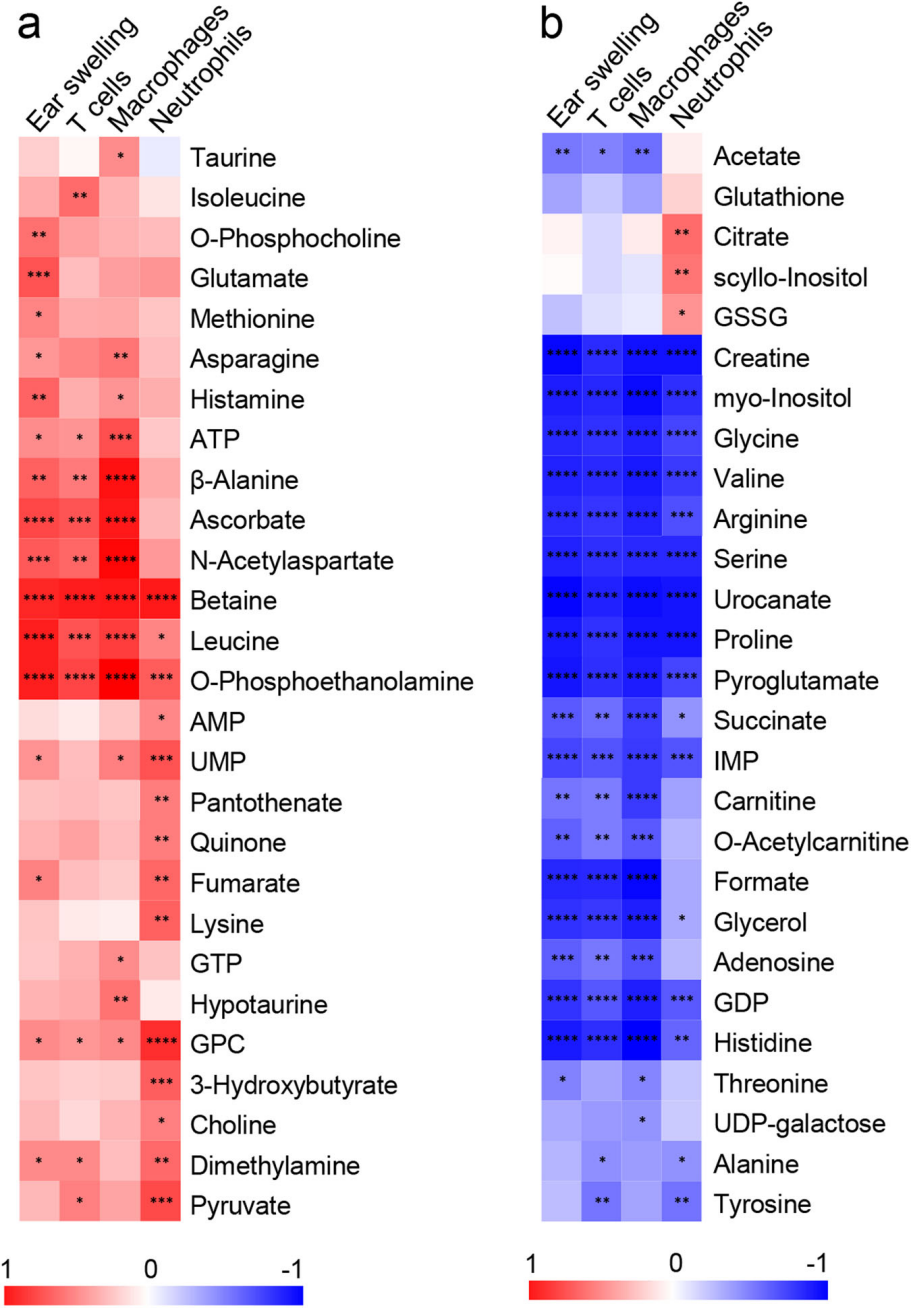
Ear swelling and immune parameters correlated to the metabolite concentrations during acute and chronic cutaneous DTHR. Ear swelling thickness, T cell (CD3), macrophage and neutrophil epidermal inflammation scores were correlated to metabolite concentration changes over the whole experimental setup, showing autoscaled (−1; 1) (**a**) positive (red) and (**b**) negative (blue) correlation with correlation coefficient statistical significance *P* values: ****<0.0001, ***<0.001, **<0.01, *<0.05, based on Pearson *r* distance measure (*n* = 5 animals).

Positive correlations were observed between the degree of ear swelling response and betaine, O-phosphocholine, ascorbate, leucine, and glutamate concentrations in inflamed ears with acute and chronic cutaneous DTHR (Fig. 4a). In contrast, we observed a negative correlation between the severity of ear swelling and the metabolites creatine, myo-inositol, IMP, formate, GDP and several amino acids, such as glycine, valine, arginine, serine, proline and histidine (Fig. 4b).

Enhanced neutrophil recruitment showed a distinctive metabolic signature compared to the degree of ear swelling as well as to the other leucocyte subtypes. In fact, increased concentrations of AMP, 3-hydroxybutyrate, choline, pantothenate, quinone, lysine, GSSG, and citrate were specifically associated with neutrophil infiltration, while the opposite trend was observed for ATP, β-alanine, ascorbate and N-acetylaspartate (NAA), which did not correlate with enhanced neutrophil recruitment (Fig. 4a).

Regarding negative correlations (Fig. 4b), carnitine, O-acetylcarnitine, formate, adenosine and acetate were negatively associated with all inflammatory variables except for neutrophil infiltration. Taurine, hypotaurine and GTP were positively associated only with macrophages. In addition, the abundance of T cells and macrophages was negatively correlated with succinate, carnitine, O-acetylcarnitine, formate, glycerol, adenosine, and acetate concentrations in inflamed ears with acute and chronic cutaneous DTHR (Fig. 4b).

### Acute and chronic inflammation is associated with changes in redox and immune metabolism

**Fig. 5:**
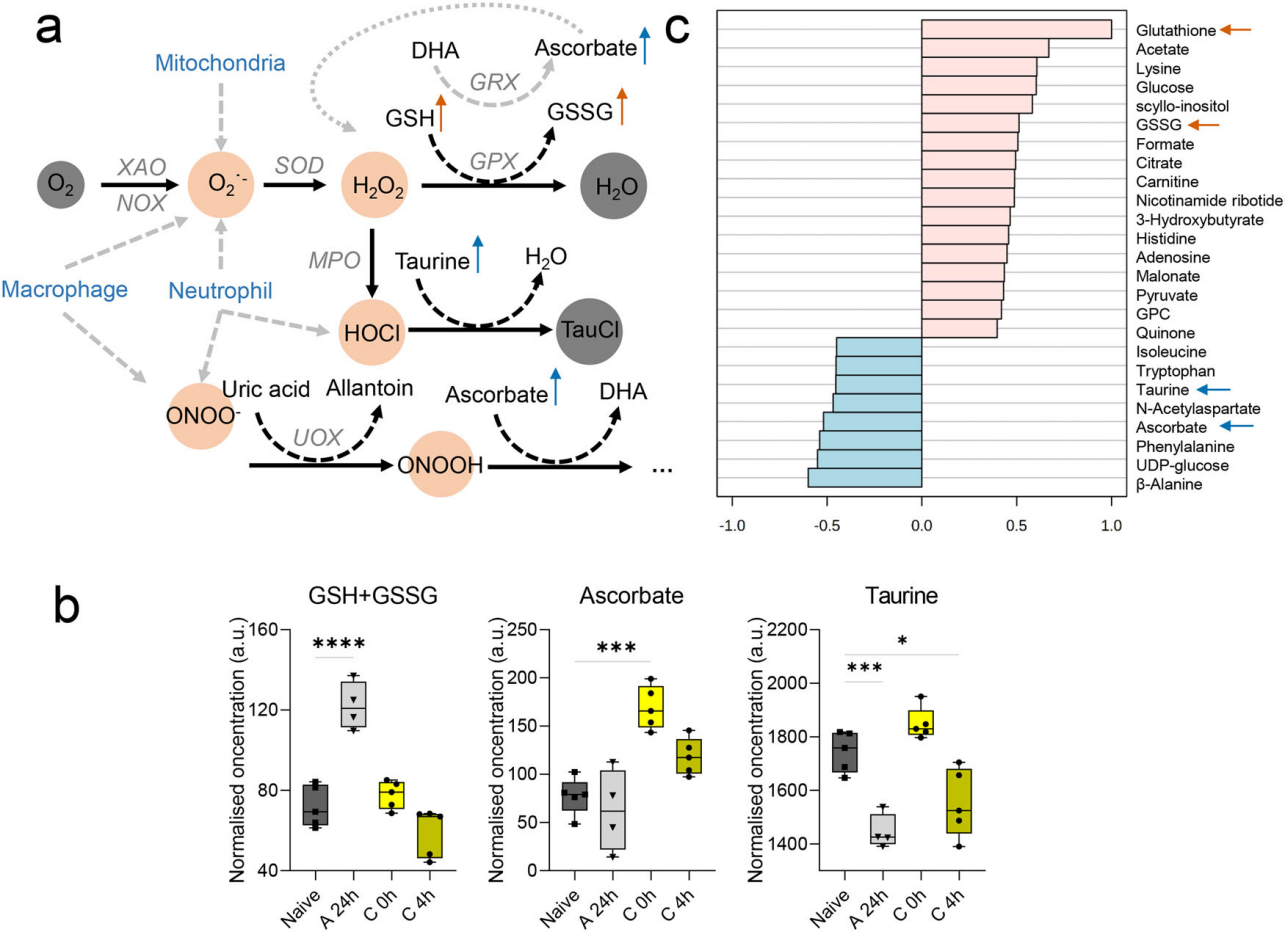
Redox and immune metabolism during acute and chronic cutaneous DTHR. **a** Overview of reactive oxygen and nitrogen species (RONS) production by resident and infiltrating immune cells during an immune response initiating the RONS cascade and their scavengers. The most important small molecule RONS scavengers, such as glutathione (GSH), ascorbate and taurine, are illustrated at their site of action. The ear swelling response during the acute cutaneous DTHR peak at 24 h is illustrated by the orange arrow, and the chronic cutaneous DTHR peak at 4 h is illustrated by the blue arrow. **b** Normalised concentration changes illustrated as box plots with max. to min. whisker, individual replicate points and median of GSH and glutathione disulfide (GSSG) concentration sum, ascorbate and taurine changes during acute 24 h (grey, downward triangle) and chronic 0 h (bright yellow, dot) and 4 h (dark yellow, dot) cutaneous DTHR (compared to naive control (grey, square), *P* values ****<0.0001, ***<0.001, **<0.01, *<0.05, one-way ANOVA). **c** Correlation pattern hunter graph of the top 25 most significantly correlated metabolites to glutathione (GSH) concentration changes over time, based on Pearson r distance measure (*n* = 5 animals, except A 24 h *n* = 4 animals). DHA dehydroascorbate, GPC sn-glycero-3-phosphocholine, GPX glutathione peroxidase, GRX glutaredoxin, GSSG glutathione disulfide, MPO myeloperoxidase, NADPH reduced nicotinamide adenine dinucleotide phosphate, NOX NADPH oxidase, SOD superoxide dismutase, TauCl taurine chloramine, UDP uridine diphosphate, UOX urate oxidase, XAO xanthine oxidase.

Redox metabolism is an interconnected network of several cascade reactions, simplified in Fig. 5a. GSH and GSSG were significantly increased during the peak of acute cutaneous DTHR, while in the chronic phase, the concentrations of GSH and GSSG were similar to the baseline measurements of healthy ears derived from naive mice (Fig. 5b and Supplementary Fig. 3a). We observed a further constant downregulation of the folate and methionine cycle metabolites serine, glycine and 5-oxoproline (pyroglutamate), which are also precursors for GSH synthesis (Supplementary Fig. 3a), in inflamed ears with both acute and chronic cutaneous DTHR.

The overall GSH and GSSG concentration pattern of inflamed ears with acute and chronic cutaneous DTHR positively correlated with acetate, glucose, and lysine, while ascorbate and taurine revealed a negative correlation together with phenylalanine, UDP-glucose and β-alanine (Fig. 5c and Supplementary Figs. 3 and 4).

Ascorbate concentrations were increased during chronic compared to acute cutaneous DTHR (Fig. 5b), similar to ATP and N-acetylaspartate (Supplementary Fig. 3b). Although the results above show significant changes in the common redox metabolites, we did not observe any significant changes in the overall NAD^+^, which is crucial for redox metabolism, over the investigated period (Supplementary Fig. 3b). Furthermore, taurine, which can act as a RONS scavenger, was consumed during acute cutaneous DTHR peaking 24 h after the 1st TNCB ear challenge (Fig. 5b). At 48 h after the 4th challenge, the point in time shortly before the 5th TNCB ear challenge, the taurine concentration within the inflamed ear was similar to that in the naive control ear, while taurine was depleted at 4 h after the 5th TNCB ear challenge, the peak in the ear swelling response of chronic cutaneous DTHR. Together with taurine, we detected increased β-alanine, betaine and hypotaurine concentrations in the chronic phase of cutaneous DTHR (Supplementary Fig. 3c). However, histidine and its downstream metabolite urocanate were significantly downregulated during the course of chronic cutaneous DTHR while histamine concentration peaked 4 h after the 5th TNCB ear challenge (Supplementary Fig. 3d).

### Inflammation and oxidative stress are associated with metabolic responses in energy, growth and nucleotide metabolism

**Fig. 6:**
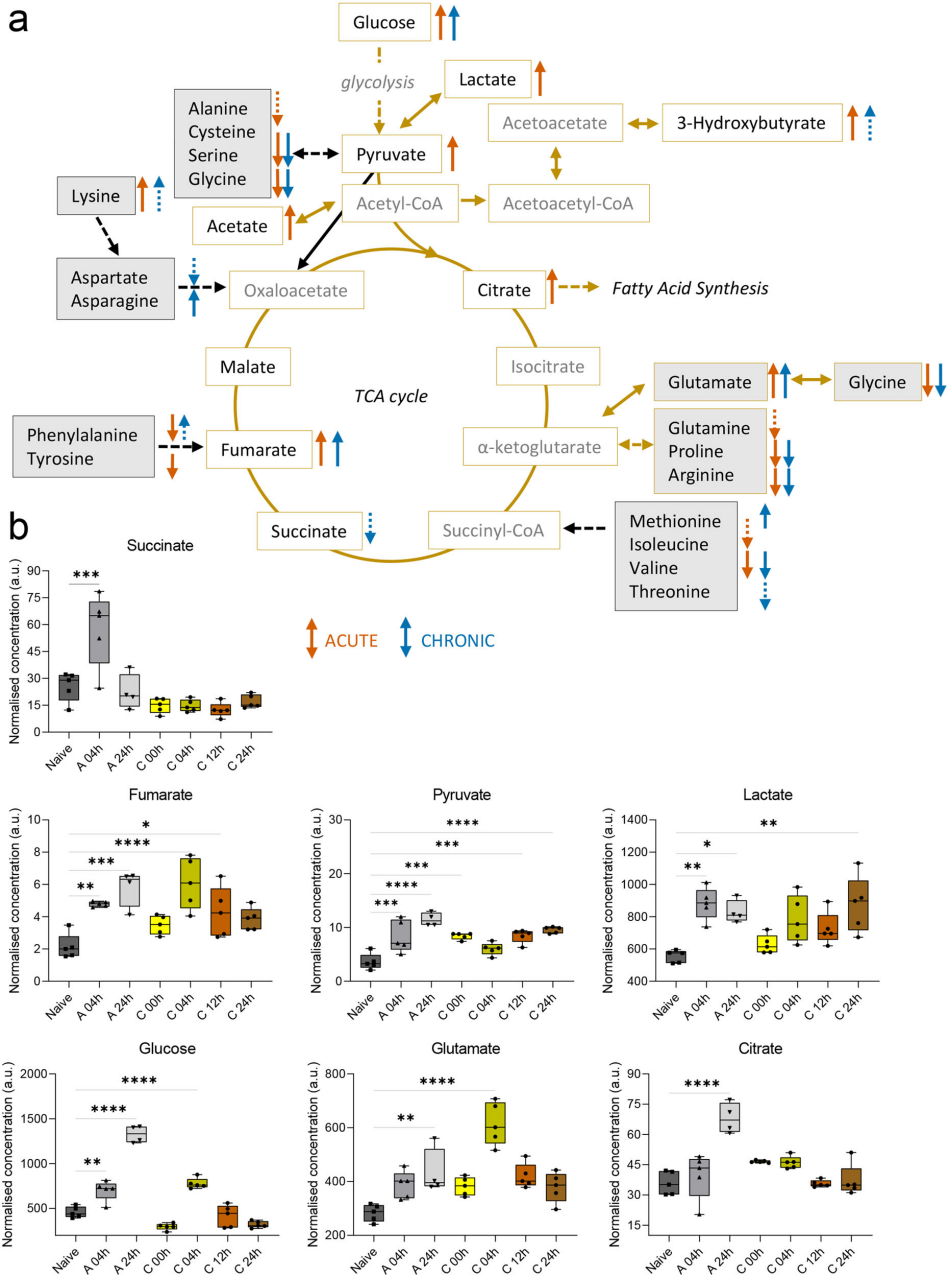
Energy metabolism during acute and chronic cutaneous DTHR. **a** Simplified illustration of the tricarboxylic acid (TCA) cycle with additional information of amino acid entry as anaplerotic (black frame, grey background) and cataplerotic substrates. Dashed line for indirect reactions with multiple steps. **b** Metabolite normalised concentration changes (in arbitrary unit (a.u.)) illustrated as box plots with max. to min. whisker, individual replicate points and median within the inflamed ears during acute 4 h (medium grey, upward triangle), 24 h (light grey, downward triangle) and chronic 0 h (bright yellow, dot), 4 h (dark yellow, dot), 12 h (orange, dot) and 24 h (brown, dot) cutaneous DTHR (compared to naive control (dark grey, square), *P* value ****<0.0001, ***<0.001, **<0.01, *<0.05, *n* = 5 animals, except A 24 h *n* = 4 animals). Statistical significance test stars shown for comparisons to naive control by one-way ANOVA. The ear swelling response during the acute cutaneous DTHR peak at 24 h is illustrated by the orange arrow, and the chronic cutaneous DTHR peak at 4 h is illustrated by the blue arrow. The dashed lines show a non-significant trend.

Next, we investigated the metabolites related to energy, growth, and cellular renewal metabolism (Fig. 6a and Supplementary Figs. 4 and 5). As early as 4 h after the 1st TNCB ear challenge, a significant accumulation of succinate was detected (Fig. 6b). Furthermore, we quantified increased concentrations of pyruvate, lactate, fumarate, glutamate, citrate, and glucose that peaked in inflamed ears with acute cutaneous DTHR 24 h after the 1st TNCB ear challenge. Meanwhile, anaplerotic TCA cycle substrates of fumarate (phenylalanine and tyrosine), succinyl-CoA (isoleucine, valine) and pyruvate (serine, glycine, alanine) were downregulated in inflamed ears with acute cutaneous DTHR compared to naive control ears (Supplementary Figs. 3 and 4). Most of these metabolites were permanently downregulated without recovering their baseline concentrations even in the chronic state of cutaneous DTHR. Furthermore, cataplerotic substrates of α-ketoglutarate (arginine, proline) showed similar concentration decay in inflamed ears with initially acute but also with chronic cutaneous DTHR. Creatine was similarly consumed as its metabolic precursors arginine, proline, glycine and serine, once again stressing the downregulation of these metabolic pathways during chronic cutaneous DTHR (Supplementary Fig. 4a). In addition, the moderate irritative toxic peak of the ear swelling response at 4 h after the 1st TNCB ear challenge was further characterised by the build-up of choline, GPC and UMP (Supplementary Fig. 5a, b). Conversely, we observed decay of IMP and GDP in inflamed ears with chronic cutaneous DTHR (Supplementary Fig. 5c).

During chronic cutaneous DTHR, an accumulation of phenylalanine, tyrosine and glutamate was observed 4 h after the 5th TNCB ear challenge, suggesting disrupted substrate uptake (Supplementary Fig. 4a) at the peak of chronic inflammation. We also identified an increase in the fumarate and glucose concentrations at 4 h during chronic cutaneous DTHR (Fig. 6). Similarly, we observed an increase in methionine (Supplementary Fig. 3), isoleucine, alanine, lysine, asparagine concentrations, glutamate/serine ratio (Supplementary Fig. 4a, b), O-phosphocholine and O-phosphoethanolamine concentrations (Supplementary Fig. 5b), while myo-inositol and glycerol concentrations decreased during chronic cutaneous DTHR (Supplementary Fig. 5d).

## Discussion

We identified specific metabolite patterns for ear swelling and the immune response during acute and chronic cutaneous DTHR. Acute cutaneous inflammation was associated with early succinate accumulation, disrupted energy metabolism with alterations in the TCA cycle, increased glutathione metabolism, and a switch to ketone body production and β-oxidation, while the chronic phase is characterised by increased ascorbate and taurine as RONS scavengers, indicating a switch in redox metabolism. Both types of inflammation showed alterations in metabolic precursors of membrane lipid synthesis, turnover and the Kennedy pathway. These metabolite signatures correlated well with the characteristic inflammatory response peaks of acute DTHR at 24 h after the 1st TNCB ear challenge and chronic cutaneous DTHR at 4 h after the 5th TNCB challenge, as reported in the previous studies^8,9^. Our data confirm that acute and chronic inflammation leads to differences in the immune cell composition, illustrated by the changes in the concentration of metabolites involved in redox, immune, energy, cell growth and nucleotide metabolism.

Resident macrophages are the predominant immune cell type in naive ear tissue, also previously shown in a psoriatic mouse model^24^. Acute inflammation is characterised by a strong influx of neutrophils and activation of resident macrophages; however, this influx of neutrophils peaks at 24 h post 1st challenge, as discussed in the previous work^9^. Changes observed in samples collected 4 h post 1st TNCB challenge reflect the innate immune response of the resident cells, as no infiltrated immune cells were detected at this time. Furthermore, acute inflammation is accompanied by strong oedema and neutrophil recruitment, resulting in increased ear swelling. In our dataset, we quantified a significant elevation of succinate 4 h after the 1st TNCB ear challenge during acute inflammation. Succinate accumulation reportedly signifies a disruption in the TCA cycle^25^. This disruption was later confirmed by subsequent citrate, pyruvate, lactate and fumarate accumulation at the acute phase 24 h after the 1st TNCB challenge. Succinate is also a pleiotropic signalling molecule responsible for HIF-1α stabilisation and continuation of proinflammatory signalling^26^. Succinate was further reduced to baseline 24 h after the 1st challenge and remained at low levels throughout the course of chronic inflammation, further confirming its unique role as an early inducer in the innate immune reaction.

GSH is a major physiological antioxidant, and it plays a crucial role in redox signalling and inflammatory immune cascades. GSH controls the innate immune response in the context of infection^25^. Amino acids such as serine, glycine and threonine are precursors for the folate cycle, while glycine, together with cysteine and glutamate, is also a precursor for GSH synthesis. We quantified the highest GSH and GSSG concentrations at the acute inflammatory response peak 24 h after the 1st TNCB challenge, correlating to the infiltrating neutrophil peak.

Short-chain fatty acids have been reported in inflammation-regulatory processes as anti-inflammatory agents^27,28^. We quantified acetate upregulation during the acute cutaneous DTHR, which was later reduced to baseline levels in the chronic phase of cutaneous DTHR, similar to the naive control. Acetate concentration changes negatively correlated with macrophage and T-cell infiltration patterns during acute and chronic inflammation progression.

Furthermore, the 3-hydroxybutyrate concentration was increased in the acute phase of cutaneous DTHR, indicating a response to increased energy consumption and reduced fatty acid stock^29^. Switching to alternative energy substrates, such as fatty acid β-oxidation, can be seen as a result of a decreased TCA cycle, as indicated by the accumulation of the TCA cycle-related metabolites fumarate, pyruvate, lactate, glutamate, citrate and even glucose during the acute inflammation peak.

The histopathological analysis confirmed that acute inflammation was dominated by the infiltration of neutrophils, while chronic cutaneous DTHR was associated with an influx of T cells, a dense accumulation of macrophages, hyperkeratosis and acanthosis. The influx and activation of immune cells peaked at 4 h after the 5th TNCB ear challenge, as shown previously^9^. Different leucocytes are recruited to the site of inflammation, including macrophages, CD4^+^ and CD8^+^ T cells, B cells and neutrophils. Resident macrophages and mast cells proliferate during chronic inflammation. While neutrophils are still present, their percentage is lower than that in the acute state due to the presence of other recruited cell types confirmed by immunohistochemistry. Neutrophils highly express NADPH oxidase 2 (NOX2), producing excess superoxide, which is quickly converted to hydrogen peroxide (H_2_O_2_) and further downstream to hypochlorous acid (HOCl) by MPO. Furthermore, the iNOS enzyme facilitates nitric oxide (NO) production, which is further converted to peroxynitrite (ONOO^−^)^30^. Moreover, fibrosis, angiogenesis, an increase in connective tissue and vascularisation are additional consequences of a chronic inflammatory state^31^.

We observed increased ascorbate and taurine 48 h after the 4th challenge, the time point of the 5th TNCB ear challenge, which was reduced at 4 h after the 5th TNCB ear challenge. GSH and GSSG concentrations in inflamed ears were restored back to similar concentrations as those determined in naive control ears. In mouse liver, GSH is consumed during oxidation to produce ascorbate from dehydroascorbate (DHA)^32,33^. This reaction, however, also produces additional hydrogen peroxide (H_2_O_2_). However, in humans, ascorbate cannot be synthesised, though it still participates in RONS scavenging^34^.

In addition, serine was depleted during chronic cutaneous DTHR. A previous report demonstrated that supplementation with serine can re-establish methionine cycle activity and GSH synthesis due to a shift from glycolysis to GSH production and one-carbon metabolism^35^. Therefore, depletion of serine could lead to the downregulation of the methionine cycle and eventually to GSH depletion during the chronic phase of cutaneous DTHR.

Taurine, a common HOCl scavenger^36^ that accumulates in tissues exposed to oxidative stress^37^, was consumed during the acute phase, thereby negatively correlating to the GSH and GSSG concentrations. Taurine build-up has been previously quantified in M1-type macrophages when GSH is depleted^38^, potentially indicating the redox-dependent balance between the two scavengers. In addition, alterations of the taurine concentration have been previously reported in psoriatic skin lesions^39^, similar to our studies related to oxidative stress and ROS scavenging well correlating to the alterations in taurine activity within inflamed ears during the course of chronic DTHR.

Furthermore, β-alanine was highly upregulated in inflamed ears during chronic cutaneous DTHR. β-Alanine is linked to the activation of T and B cells^40^. The progression from acute to chronic inflammation in our model is associated with increased infiltration of T and B cells in the inflamed ears could explain the increased concentrations of β-alanine. A study in a cardiac model stated that β-alanine and taurine oppose each other, indicating that β-alanine caused taurine level reduction, mitochondrial superoxide accumulation, and eventual apoptosis and reduced ATP generation^41^. This fact is consistent with our observations of increased ATP in inflamed ears with chronic cutaneous DTHR when compared to inflamed ears with acute cutaneous DTHR or naive control ears.

Together with ascorbate, N-acetylaspartate (NAA) was increased at 48 h after the 4th TNCB ear challenge, the time point shortly before the 5th TNCB ear challenge, possibly as a consequence of a metabolic adaptation mechanism. NAA was then consumed at the peak of chronic cutaneous DTHR 4 h after the 5th TNCB ear challenge for a short period with a trend of regeneration until 24 h. NAA is believed to mainly be a central nervous system-characteristic metabolite; however, it is linked to de novo lipogenesis and plays an important role in histone acetylation^42,43^. NAA accumulation has also been previously observed in tumours^42^, linking the inflammatory process to further cancer development.

Energy consumption, RONS production and cell influx are highly increased due to the progression of inflammation. RONS production during the oxidative burst leads to a switch from oxidative phosphorylation to aerobic glycolysis^44^, also known as the Warburg effect. Peaks of the ear swelling response are characterised by maximal oedema and dilated blood vessels. Glucose is one of the most abundant blood metabolites. The glucose concentration increases when the blood vessels expand. We observed an increased concentration of glucose at peaks of the acute and chronic inflammatory immune response (acute, 24 h; chronic, 4 h), followed by its rapid consumption in the later phase of chronic inflammation. Meanwhile, glutamine, which is the most abundant amino acid in the blood, was not significantly changed during the chronic cutaneous DTHR, potentially due to its multiple roles and involvement in several metabolic pathways.

Lactate accumulation was observed during both acute and chronic cutaneous DTHR. During acute cutaneous DTHR, we also determined an accumulation of pyruvate and succinate, indicating disruption of the TCA cycle and the preferential use of glucose in aerobic glycolysis. During chronic cutaneous DTHR, we observed the consumption of pyruvate at the peak of chronic inflammation (chronic, 4 h) relative to 0 h, 12 h and 24 h after the 5th TNCB ear challenge. Lactate concentrations in inflamed ears were increased, potentially suggesting pyruvate conversion to lactate due to the inflammatory peak and necessary acidic environment stabilisation for recruitment of additional inflammatory cells^45^. Consistent with the changes in TCA metabolites, a slight increase in ATP consumption was determined at 4 h after the 1st TNCB ear challenge (acute cutaneous DTHR), while the energy pool was later restored upon chronic cutaneous DTHR.

Disruption of the TCA cycle during acute cutaneous DTHR was further suggested by the downregulation of its anaplerotic substrates^46^. Some amino acids, such as phenylalanine, methionine and asparagine, further accumulated in the chronic phase. Phenylalanine accumulation has been previously linked to an acute inflammatory state due to disrupted metabolism^47,48^. However, in our dataset, we quantified the reduction in phenylalanine upon acute cutaneous DTHR, while it tended to increase during the peak of chronic cutaneous DTHR at 4 h after the 5th TNCB ear challenge.

The effector phase of TNCB-induced cutaneous DTHR does not entirely reflect the pathology of clinical allergic contact dermatitis as the T-cell-driven immune response is associated with pronounced immigration of neutrophils, including subepidermal abscesses which are not evident in human contact dermatitis. However, TNCB-induced cutaneous DTHR shares a lot of similarities with clinical psoriasis vulgaris, an antigen-specific T-cell-driven autoimmune disease.

Psoriatic skin lesions have been characterised as highly metabolically active with upregulated cell proliferation^48^. Phenylalanine, glutamate concentrations and glutamate/serine ratios were upregulated in lesional skin when compared to nonlesional skin or the skin of healthy patients^49^, which is consistent with our findings in the preclinical acute and chronic DTHR model.

Moreover, glucose, myo-inositol and branched-chain amino acid (BCAA, valine-leucine/isoleucine) levels were reduced in psoriasic skin lesions compared to nonlesional skin^49^. In our study, inflamed ears displayed increased glucose concentrations during acute and chronic inflammation, peaking at 24 h after 1st TNCB challenge and 4 h after 5th TNCB ear challenge. However, we also observed strongly reduced glucose levels during chronic cutaneous DTHR reaching baseline levels determined in healthy ears. Within our studies, myo-inositol and valine followed the trend described in human psoriasis with a significant reduction during both acute and chronic DTHR^49^.

We also observed alterations in the histidine metabolism during chronic cutaneous DTHR. Thus, histidine and urocanate in inflamed ears were significantly downregulated while histamine concentration peaked 4 h after the 5th TNCB ear challenge. In line with our data, alterations in histidine metabolism have been previously reported in the context of human psoriasis skin inflammation, highlighting the histidine metabolic pathway as a potential therapeutic target^50^.

We observed increased tryptophan consumption during the peak of acute cutaneous DTHR at 24 h after the 1st TNCB ear challenge as a potential indicator of the anti-inflammatory capacity of the system. Tryptophan has been linked to the immune response via the kynurenine pathway^51^, further connecting chronic inflammation to downstream systemic effects, such as gut dysbiosis and autoimmune disease^52^. Its degradation is a marker for immune-regulatory effects toward reduced lipid peroxidation^53^, antioxidant activity^54^, antimicrobial activity of skin inflammation^55^.

Proinflammatory cytokines, which are upregulated during inflammation progression, induce changes in phospholipid species together with overall mitochondrial mass reduction^17^. While we quantified increased choline and GPC during acute cutaneous DTHR, they were restored to baseline levels during chronic cutaneous DTHR, leading to a build-up of O-phosphoethanolamine. These glycerophospholipid changes have been associated with pro- and anti-inflammatory activity via plasmalogen hydrolysis, cell membrane property changes, and inflammatory cascade triggering^56^. A switch from choline to ethanolamine metabolism has been previously shown as part of rapid cell growth and repair in environments such as tumour^57,58^. Moreover, a recent study by Fuchs et al.^38^ showed a similar build-up of O-phosphoethanolamine in M1 macrophages, resulting in reduced mitochondrial respiration and a reduced inflammatory response^38^.

Overall, ^1^H-NMR spectroscopy-based metabolomics investigation has been shown to be a useful tool for a deeper metabolic characterisation of the immune response during acute and chronic T-cell-driven inflammation. Our findings of GSH and GSSG alterations suggest a systemic response to local inflammation, as GSH is mainly synthesised in the liver^59^. Because acute inflammation is driven by resident cells and infiltrating neutrophils, while the chronic phase is characterised by an increased accumulation of T cells and macrophages, we could correlate common metabolites acting as RONS scavengers to the corresponding immune cell infiltration relative to the inflammation progression stage. Fuchs et al.^38^ in vitro studies recently reported that changes in taurine and tryptophan concentrations were associated with the activation state of macrophages^38^, supporting the results of our immune cell–metabolite correlation analysis. Meanwhile, an ex vivo study by Chokesuwattanaskul et al.^60^ has previously connected alterations in N-acetylaspartate concentrations to the activation state of neutrophils^60^. These findings are also relevant towards a better understanding of how different inflammatory stages can be involved in the tumour-promoting inflammatory response and how immunometabolomics could emerge as a novel diagnosis and therapy monitoring tool.

## Methods

### Animals

C57BL/6J mice were purchased from Jackson Laboratory (Bar Harbor, USA). Eight-week-old female mice were used for in vivo experiments. All mice were held in individually ventilated cages under the same conditions in the animal housing facility at Werner Siemens Imaging Center, Eberhard Karls University of Tübingen. This study received an ethical approval and experimental protocol approval by the German Regional Authority Committee of Tübingen (Regierungspraesidium Tübingen).

### Cutaneous delayed-type hypersensitivity reaction (DTHR) and ear sample collection

Mice were sensitised with 5% trinitrochlorobenzene (TNCB) at the abdomen and challenged after 1 week on the right ear with 1% TNCB to elicit acute DTHR (*n* = 5). Chronic DTHR was induced by repetitive challenges with 1% TNCB every 2 days up to five times (1st TNCB challenge: Day 7; 2nd: Day 9; 3rd: Day 11; 4th: Day 13, 5th: Day 15, Fig. 1a). Mice were anaesthetised by inhalation of isoflurane-O_2_ (1.5% Forane, Abbott GmbH, Wiesbaden, Germany), and the ear thickness was measured with a micrometre (Kroeplin, Schlüchtern, Germany) before (naive), 4 h, 12 h and 24 h after the 1st and 5th TNCB challenge to monitor the course of the ear swelling response.

Ear tissue (*n* = 5) was cut with scissors immediately after sacrificing experimental mice and snap-frozen in liquid nitrogen at the following time intervals (Fig. 1a): (1) naive, immediately before the 1st TNCB challenge; (2) acute inflammation: at 4 h and 24 h after the 1st TNCB challenge, referred to as A 4 h and A 24 h, respectively; (3) chronic inflammation: at 48 h after the 4th and at 4 h, 12 h and 24 h after the 5th TNCB challenge, referred to as C 0 h, C 4 h, C 12 h, and C 24 h, respectively.

### Histopathology and immunohistochemistry

Ear tissue samples (*n* = 4) were fixed in 4.5% formalin and further embedded in paraffin. Three to five micrometre-thick sections were cut from distinct samples and stained with haematoxylin and eosin (H&E) for histology. Immunohistochemistry was performed with an automated immunostainer (Ventana Medical Systems, Inc., Oro Valley, USA). The slides were stained with antibodies against the cluster of differentiation 3 protein complex (CD3, Clone SP7, DCS Innovative Diagnostik-Systeme GmbH u. Co. KG, Hamburg, Germany), myeloperoxidase (MPO, Anti-Myeoloperoxidase Ab-1, Lab Vision UK, Ltd., Newmarket, Suffolk) and ionised calcium-binding adaptor molecule 1 (Iba1, Abcam, Cambridge, UK). Appropriate positive and negative controls were used to confirm the adequacy of the staining. Photomicrographic images were acquired with an Axioskop 2 plus Zeiss microscope with a Jenoptik ProgRes C10 plus camera and software (Laser Optik System, Jena, Germany). Final image preparation was performed with Adobe Photoshop CS6.

The epidermal inflammation score was based on the presence and number of epidermal abscesses and crusts per section (0 = no damage, 1 = presence of abscesses, 2 = between 1 and 5 crusts, 3 = between 6 and 10 crusts, 4 = more than 11 crusts). In addition, a semiquantitative analysis of dermal inflammation was performed (“−” = no inflammatory infiltrate, “+” = minimal inflammatory cells, “++” = mild inflammation, “+++” = moderate inflammation, “++++” = severe amount of inflammatory cells). Due to a technical issue, one sample of the chronic 12 h group had to be excluded from further analysis. Therefore, chronic 12 h group *n* = 3 animals, while all the other sample conditions, naive, acute 4 h and 24 h, chronic 0 h, 4 h, and 24 h were *n* = 4 animals.

### Ear tissue preparation for metabolomics

Deep-frozen ear tissue (*n* = 5) was cryogenically pulverised (Covaris cryoPREP CP02, Woburn, MA, USA) using liquid nitrogen and transferred to adaptive focused acoustics (AFA)-compatible glass tubes. A two-phase metabolite extraction protocol was applied to remove lipid signals from the ^1^H-NMR spectra. Therefore, 300 µL of methanol (LC–MS grade, Sigma Aldrich, Taufkirchen, Germany) and 1000 µL of *tert*-butyl methyl ether (MTBE, Sigma Aldrich, Taufkirchen, Germany) were added to the homogenous tissue powder and gently vortexed. A 5 min ultrasonication programme was employed for metabolite extraction (Covaris E220 Evolution, Woburn, MA, USA). Then, 250 µL of ultrapure water was added for two-layer phase separation. After dividing the layers, the polar phase was evaporated to dryness and used for subsequent metabolomics analysis.

### Sample preparation and ^1^H-NMR spectroscopy acquisition

Dry metabolite pellets were resuspended in a deuterated phosphate buffer (pH corrected to 7.4) containing 1 mM 3-(trimethylsilyl) propionic-2,2,3,3-d_4_ acid sodium salt (TSP) as an internal standard (Sigma Aldrich Chemie, Taufkirchen, Germany). NMR spectra were recorded from distinct samples at 298 K on a 14.1 Tesla ultrashielded NMR spectrometer (600 MHz proton frequency, Avance III HD, Bruker BioSpin, Ettlingen, Germany) equipped with a triple resonance 1.7 mm room temperature microprobe. Short zero-go (zg), 1D nuclear Overhauser effect spectroscopy (NOESY) and Carr-Purcell-Meiboom-Gill (CPMG, 1024 scans) pulse programmes were used for spectral acquisition. Bruker TopSpin 3.6.1 software was used for spectral preprocessing, and ChenomX NMR Suite 8.5 was used for metabolite assignment and quantification. Supplementary Fig. 2 illustrates metabolite spectra assignment examples.

### Statistics and reproducibility

Most statistical analyses were performed with the MetaboAnalyst 5.0 web server^61^. Here, data were normalised for dilution effects by the probabilistic quotient normalisation (PQN) approach on a reference sample. Heatmaps were produced using non-parametric one-way ANOVA, Fischer’s least significant difference (LSD) was applied in the post hoc analysis, and unsupervised clustering was achieved by the Ward clustering algorithm employing the Euclidean distance measure. Heatmaps show autoscaled and normalised metabolite concentration values. For visualisation and further statistical analysis, we used Graph Pad Prism (Version 9.1.0, Graph Pad Software, San Diego, CA, USA) and R version 1.4.11.06. The individual metabolite concentrations were visualised as box plots with max. to min. whiskers with individual data points, and *P* values illustrated statistically significant metabolites compared to the naive baseline control: ****<0.0001, ***<0.001, **<0.01, *<0.05.

Due to the technical issue of poor spectral resolution, one sample of the acute 24 h group had to be excluded from further analysis. Therefore, acute 24 h is *n* = 4 animals, while all the other sample conditions, naive, acute 4 h, chronic 0 h, 4 h, 12 h and 24 h were *n* = 5 animals. The number of replicates was defined by minimum necessary replicates for metabolomics studies for efficient statistical power of metabolite concentration data and available number of animals as defined by the ethical approval.

### Reporting summary

Further information on research design is available in the Nature Portfolio Reporting Summary linked to this article.

## Supplementary information

Supplementary Information

Description of Additional Supplementary Files

Supplementary Data 1

Supplementary Data 2

Supplementary Data 3

Reporting Summary

## Data Availability

Supplementary data with the source data are provided. Supplementary Data 1: ear thickness measurement data for Fig. 1b dot plot. Supplementary Data 2: normalised source data for metabolite dot/box plots, heatmaps, correlation graphs, and principal component analysis. Supplementary Data 3: the source data for Fig. 3 histopathology scoring and correlation analysis. ^1^H-NMR spectra data are available upon request.
