# Supplementary material for: Acute and chronic inflammation alter immunometabolism in a cutaneous delayed-type hypersensitivity reaction (DTHR) mouse model
Source: Commun Biol. 2022 Nov 15;5:1250. doi: 10.1038/s42003-022-04179-x (PMC9666528; doi:10.1038/s42003-022-04179-x)
Supplement: Supplementary file 1 — Supplementary Information [file 42003_2022_4179_MOESM1_ESM.pdf]

Supplementary Figures

Acute and chronic inflammation alter immunometabolism in a cutaneous delayed-type hypersensitivity reaction (DTHR) mouse model

Laimdota Zizmare[1], Roman Mehling[1], Irene Gonzalez-Menendez[2,3], Caterina Lonati[4], Leticia Quintanilla-Martinez[2,3], Bernd J. Pichler[1,2], Manfred Kneilling[1,2,5,#], Christoph Trautwein[1,#]

[1] Werner Siemens Imaging Center, Department of Preclinical Imaging and Radiopharmacy, Eberhard Karls University of Tübingen, Röntgenweg 13, 72076, Tübingen, Germany
[2] Cluster of Excellence iFIT (EXC 2180) "Image-Guided and Functionally Instructed Tumor Therapies", Eberhard Karls University of Tübingen, Röntgenweg 11, 72076, Tübingen, Germany
[3] Institute of Pathology and Neuropathology, Comprehensive Cancer Center, Eberhard Karls University of Tübingen, Liebermasterstraße 8, 72076, Tübingen, Germany
[4] Center for Preclinical Research, Fondazione IRCCS Ca' Granda Ospedale Maggiore Policlinico, Via Pace 9, 20100 Milan, Italy
[5] Department of Dermatology, Eberhard Karls University of Tübingen, Liebermeisterstraße 25, 72076, Tübingen, Germany
[#]Corresponding authors: Manfred Kneilling Manfred.Kneilling@med.uni-tuebingen.de and Christoph Trautwein Christoph.Trautwein@med.uni-tuebingen.de

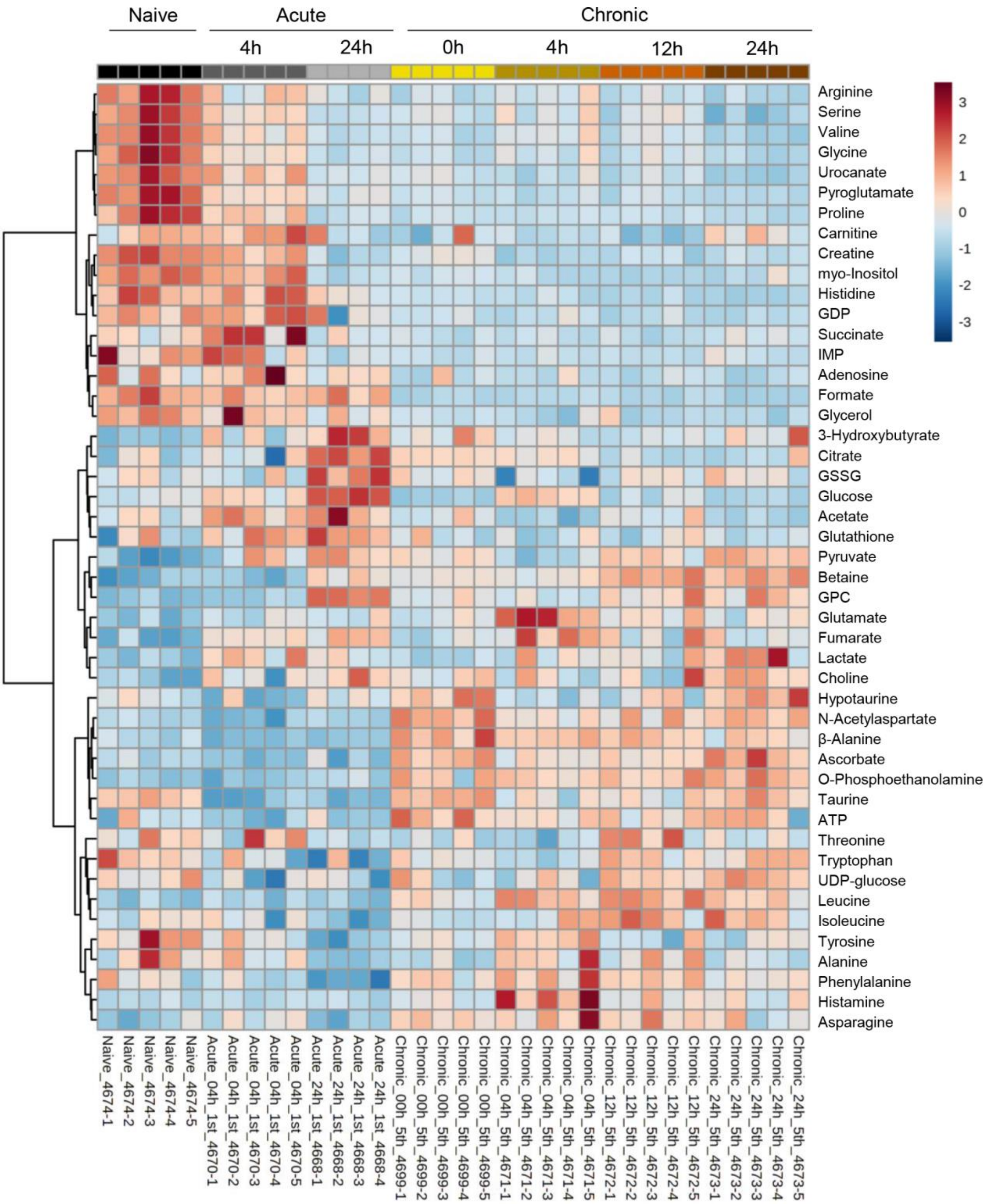

Supplementary figure 1: Overview of ^{1}H-NMR spectroscopy-based metabolomics analysis quantified metabolites during acute and chronic cutaneous DTHR. Heatmap of 54 statistically significant quantified metabolites showing each sample individual metabolite changes as auto-scaled (-4; 4) normalised concentration values, further clustered with Ward clustering method and hierarchical clustering algorithm. Red indicating relatively high concentration and blue – relatively low concentration (n=5 animals, except acute 24 h n=4 animals).

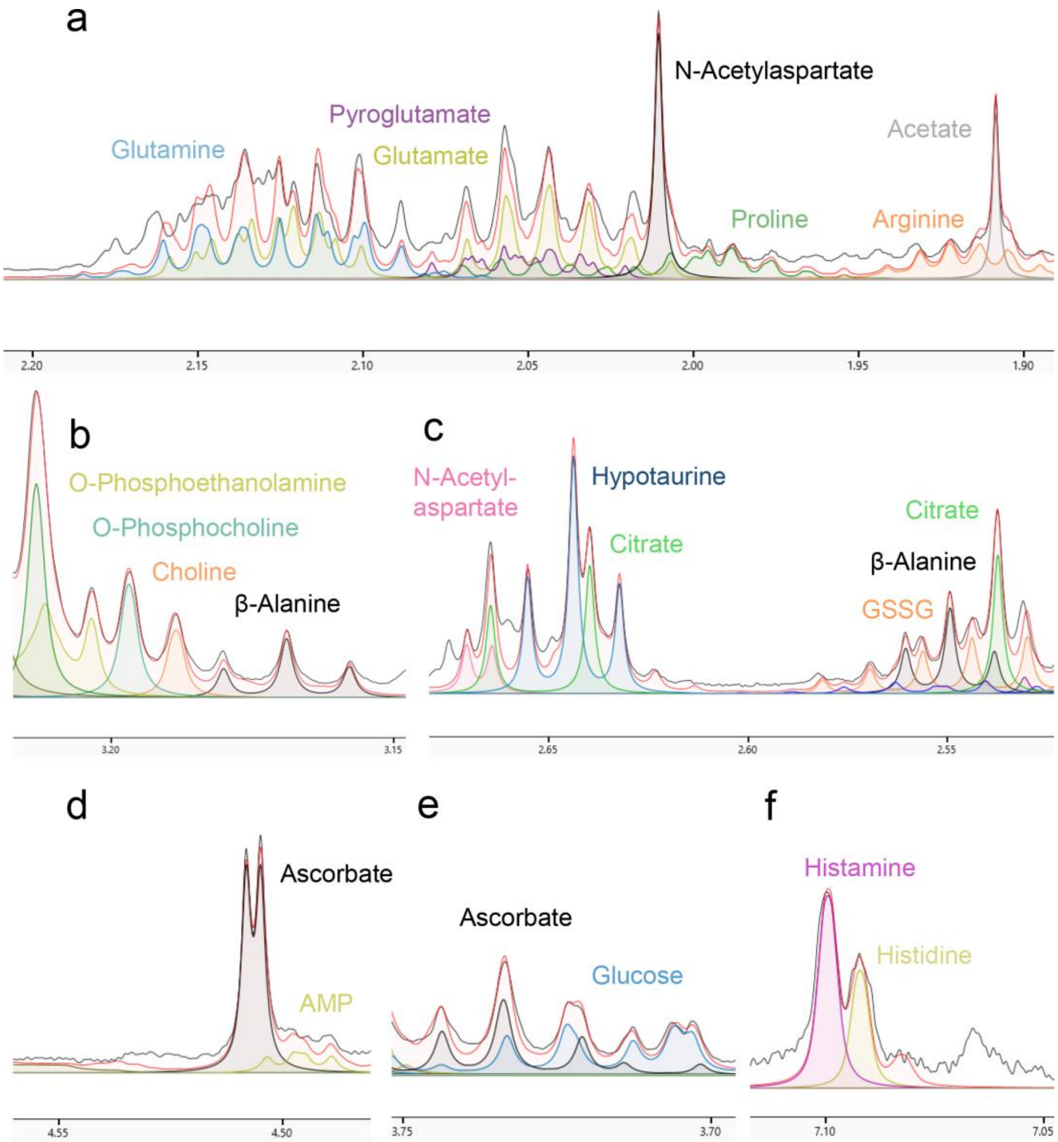

Supplementary figure 2: ^{1}H-NMR spectroscopy chronic 0 h DTHR (48 h after the 4th challenge) example spectra assignment. Example characteristic resonance regions are shown for (**a**) N-acetylaspartate, (**b**) β-alanine, (**c**) hypotaurine, (**d, e**) ascorbate and (**f**) histamine among other metabolite neighbouring peak resonances. All metabolites, that have multiple resonances in spectra were further confirmed in other regions for complete spectra assignment and quantification. Red line indicates sum spectra outline considering all overlapping peaks. Black thin line indicates original spectra. Coloured peak areas with bold outline show metabolite assignment according to the typed metabolite name in corresponding colour.

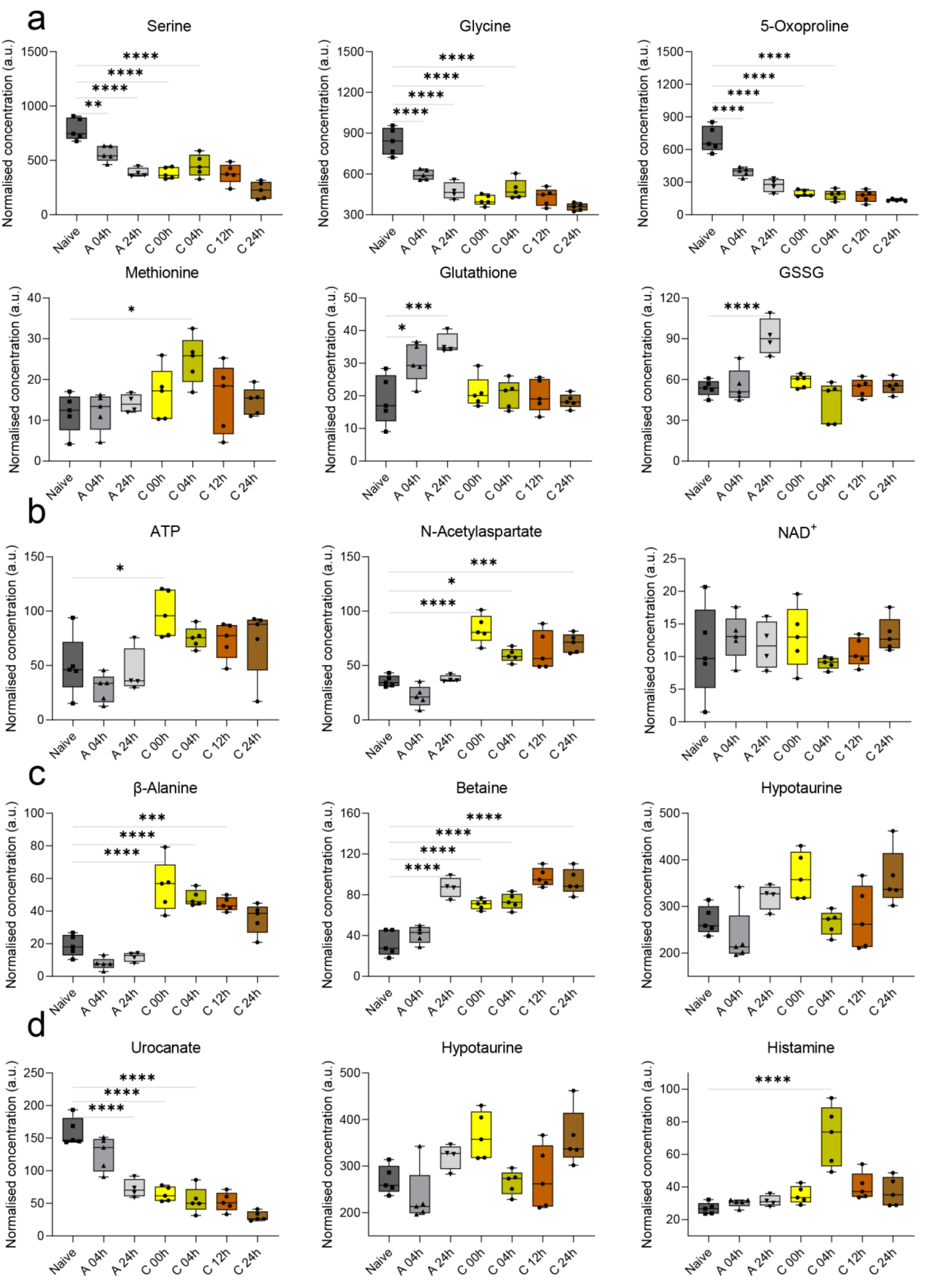

Supplementary Figure 3: Redox and immune metabolism additional metabolite box plots during acute and chronic cutaneous DTHR. (**a**) Redox-related metabolites serine, glycine and 5-oxoproline were downregulated during acute and chronic DTHR, while methionine was slightly increased at the chronic inflammation peak; glutathione and glutathione disulfide (GSSG) show increased concentrations

during acute DTHR; (**b**) ATP and N-acetylaspartate were increased during chronic DTHR, while NAD^+ concentration was not significantly affected; (**c**) taurine-related metabolites β-alanine, betaine and hypotaurine were increased during chronic DTHR; (**d**) histidine, fatty acid and galactose metabolic pathway-related metabolites urocanate and histidine were reduced during acute and chronic cutaneous DTHR while histamine peaked at chronic 4 h. Individual metabolite box plots illustrating normalised concentration in arbitrary unit (a.u.) with max. to min. whisker, individual replicate points and median with p values **** < 0.0001, *** < 0.001, ** < 0.01 * < 0.05, one-way ANOVA statistical test, n=5 animals, except acute 24 h n=4 animals). Statistically significant stars shown for comparisons to naive control.

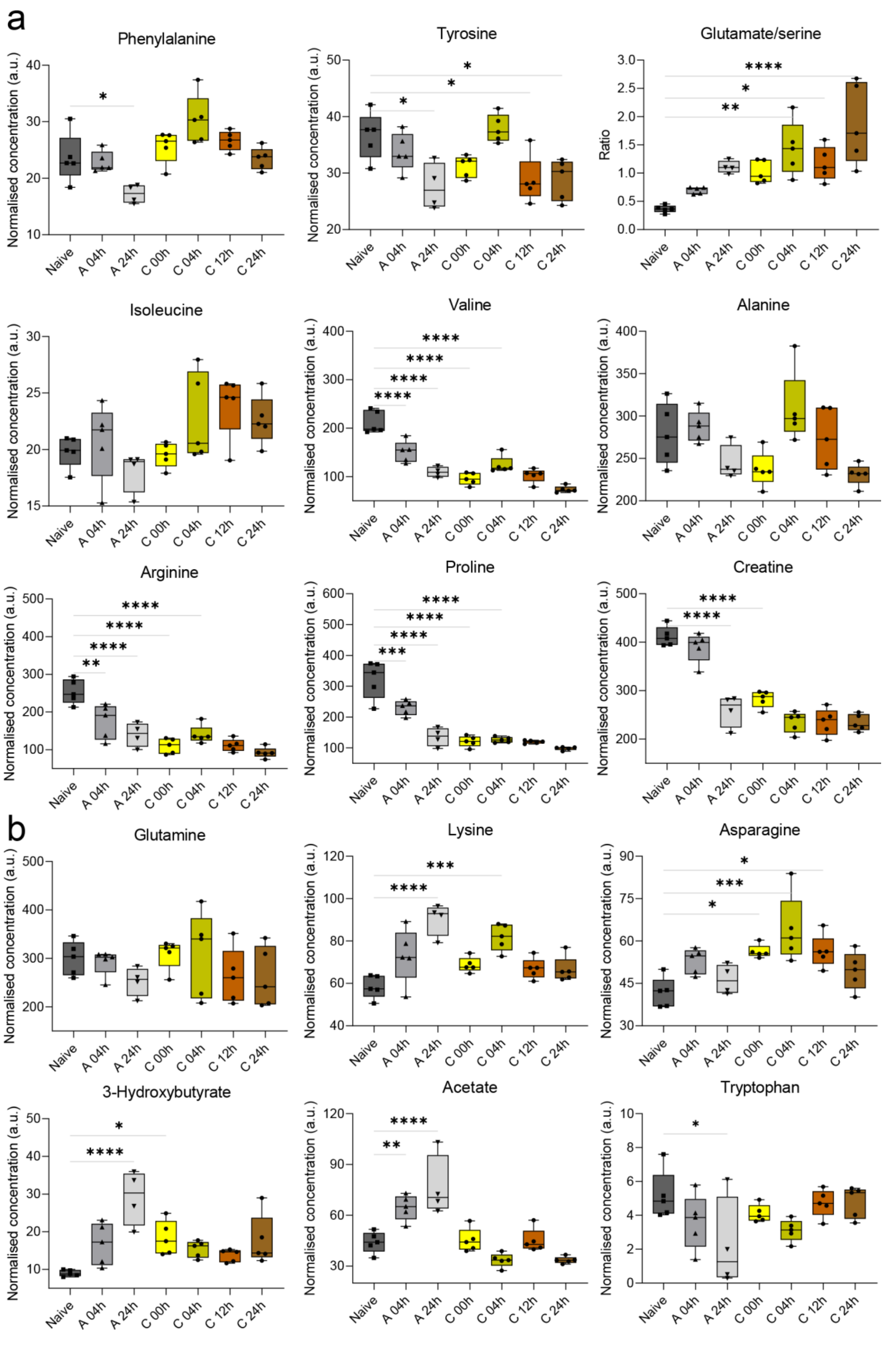

Supplementary Figure 4: Energy metabolism during acute and chronic cutaneous DTHR. (a) Phenylalanine, isoleucine, glutamate/serine ratio, tyrosine and alanine follow similar concentration

change patterns, while valine, arginine, proline and creatine concentrations are reduced during DTHR progression. (**b**) Glutamine concentrations were not significantly changed over the course of acute and chronic cutaneous DTHR; lysine, 3-hydroxybutyrare and acetate follow similar concentration change patterns; asparagine concentration increased during chronic cutaneous DTHR; tryptophan concentrations were reduced in acute and chronic cutaneous DTHR compared to the naive control. Normalised concentration changes are illustrated as box plots with max. to min. whisker, individual replicate points and median (p value **** < 0.0001, *** < 0.001, ** < 0.01 * < 0.05) (n = 5). Statistically significant stars shown for comparisons to naive control, by one-way ANOVA, n=5 animals, except acute 24 h n=4 animals.

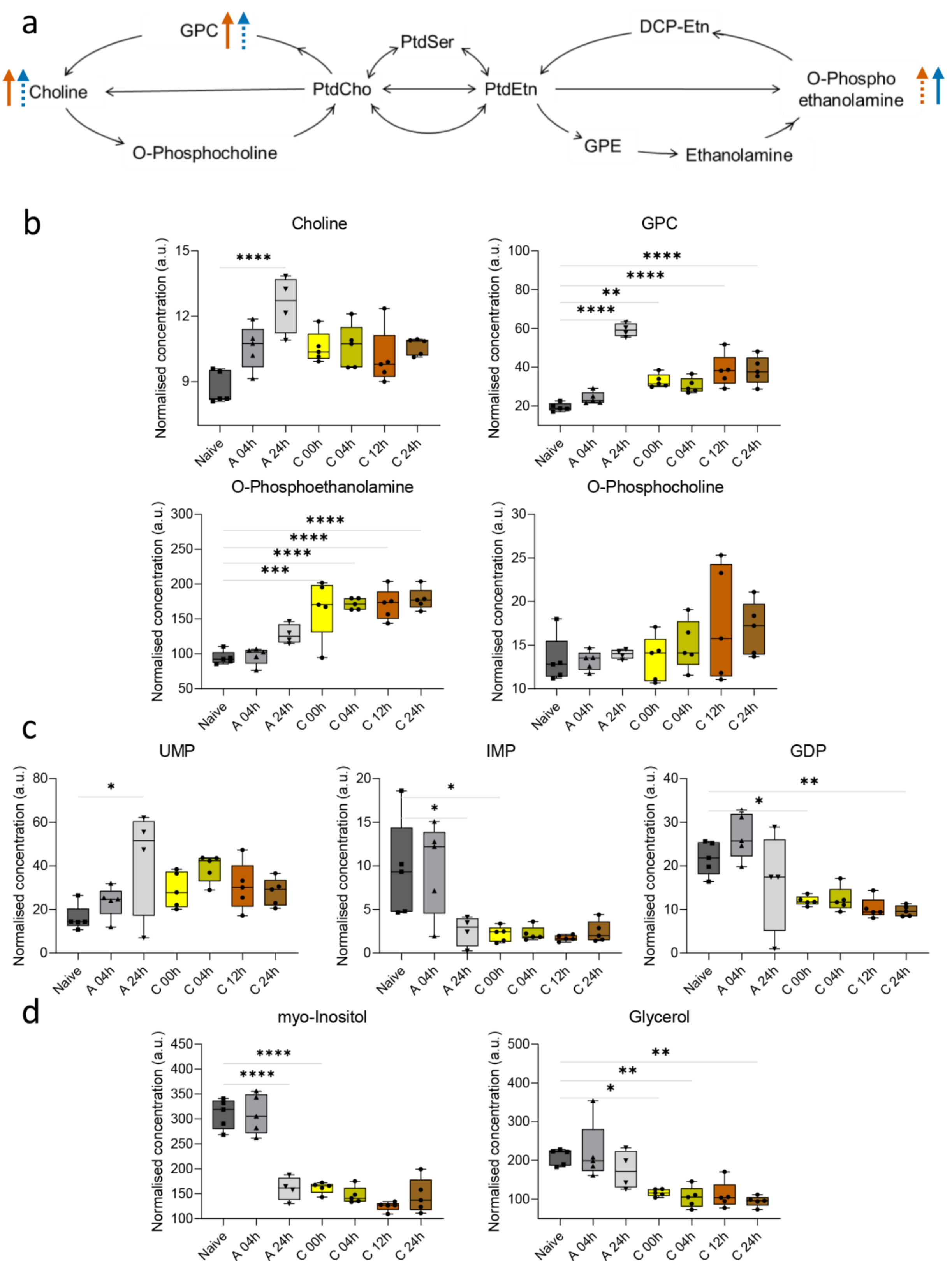

Supplementary Figure 5: Growth and nucleotide metabolism-related metabolite normalised concentration changes during acute and chronic cutaneous DTHR. (**a**) Kennedy pathway adapted from Esmaeli *et al.*, 2014; (**b**) Kennedy pathway-related metabolites; (**c**) nucleotide metabolism-related metabolites; (**d**) myo-inositol and glycerol were reduced during acute and chronic cutaneous DTHR. Concentration changes illustrated as box plots with max. to min. whisker, individual replicate points and

median (p value **** < 0.0001, *** < 0.001, ** < 0.01 * < 0.05). Statistically significant stars shown for comparisons to naive control, one-way ANOVA, n=5 animals, except acute 24 h n=4 animals. Orange arrows indicate concentration increase or decrease compared to naive control at acute inflammation peak at 24 h, blue – chronic inflammation peak at 4 h, dashed lines show a non-significant concentration trend.
