# Supplementary material for: Acute and chronic inflammation alter immunometabolism in a cutaneous delayed-type hypersensitivity reaction (DTHR) mouse model
Source: Commun Biol. 2022 Nov 15;5:1250. doi: 10.1038/s42003-022-04179-x (PMC9666528; doi:10.1038/s42003-022-04179-x)
Supplement: Supplementary file 2 — Description of Additional Supplementary Files [file 42003_2022_4179_MOESM2_ESM.pdf]

Description of Additional Supplementary Files

File name: Supplementary Data 1

Description: Ear thickness measurement data for Figure 1b dot plot.

File name: Supplementary Data 2

Description: Normalised source data for metabolite dot/box plots, heat maps, correlation graphs, and principal component analysis.

File name: Supplementary Data 3

Description: The source data for Figure 3 histopathology scoring and correlation analysis.
