# Supplementary material for: Acute and chronic inflammation alter immunometabolism in a cutaneous delayed-type hypersensitivity reaction (DTHR) mouse model
Source: Commun Biol. 2022 Nov 15;5:1250. doi: 10.1038/s42003-022-04179-x (PMC9666528; doi:10.1038/s42003-022-04179-x)
Supplement: Supplementary file 6 — Reporting Summary [file 42003_2022_4179_MOESM6_ESM.pdf]

nature portfolio

Corresponding author(s): PD Dr. Manfred Kneilling

Last updated by author(s): Sep 29, 2022

Reporting Summary

Nature Portfolio wishes to improve the reproducibility of the work that we publish. This form provides structure for consistency and transparency in reporting. For further information on Nature Portfolio policies, see our Editorial Policies and the Editorial Policy Checklist.

Statistics

For all statistical analyses, confirm that the following items are present in the figure legend, table legend, main text, or Methods section.

n/a	Confirmed	
☐	☒	The exact sample size (*n*) for each experimental group/condition, given as a discrete number and unit of measurement
☐	☒	A statement on whether measurements were taken from distinct samples or whether the same sample was measured repeatedly
☐	☒	The statistical test(s) used AND whether they are one- or two-sided<br>*Only common tests should be described solely by name; describe more complex techniques in the Methods section.*
☒	☐	A description of all covariates tested
☐	☒	A description of any assumptions or corrections, such as tests of normality and adjustment for multiple comparisons
☐	☒	A full description of the statistical parameters including central tendency (e.g. means) or other basic estimates (e.g. regression coefficient) AND variation (e.g. standard deviation) or associated estimates of uncertainty (e.g. confidence intervals)
☐	☒	For null hypothesis testing, the test statistic (e.g. F, t, r) with confidence intervals, effect sizes, degrees of freedom and P value noted<br>*Give P values as exact values whenever suitable.*
☒	☐	For Bayesian analysis, information on the choice of priors and Markov chain Monte Carlo settings
☒	☐	For hierarchical and complex designs, identification of the appropriate level for tests and full reporting of outcomes
☐	☒	Estimates of effect sizes (e.g. Cohen's d, Pearson's r), indicating how they were calculated

Our web collection on statistics for biologists contains articles on many of the points above.

Software and code

Policy information about availability of computer code

Data collection	Spectral data was acquired and processed by Bruker TopSpin 3.6.1 software. Photomicrographic histology images were acquired with an Axioskop 2 plus Zeiss microscope with a Jenoptik ProgRes C10 plus camera and software (Laser Optik System, Jena, Germany).
Data analysis	ChenomX NMR Suite 8.5 was used for metabolite assignment and quantification. MetaboAnalyst 5.0 web server metaboanalyst.ca and Graph Pad Prism (Version 9.1.0, Graph Pad Software, San Diego, CA, USA) were used for statistical analysis.

For manuscripts utilizing custom algorithms or software that are central to the research but not yet described in published literature, software must be made available to editors and reviewers. We strongly encourage code deposition in a community repository (e.g. GitHub). See the Nature Portfolio guidelines for submitting code & software for further information.

Data

Policy information about availability of data

All manuscripts must include a data availability statement. This statement should provide the following information, where applicable:

- Accession codes, unique identifiers, or web links for publicly available datasets
- A description of any restrictions on data availability
- For clinical datasets or third party data, please ensure that the statement adheres to our policy

Metabolomics dataset can be provided upon request.

Human research participants

Policy information about studies involving human research participants and Sex and Gender in Research.

Reporting on sex and gender	n/a
Population characteristics	n/a
Recruitment	n/a
Ethics oversight	n/a

Note that full information on the approval of the study protocol must also be provided in the manuscript.

Field-specific reporting

Please select the one below that is the best fit for your research. If you are not sure, read the appropriate sections before making your selection.

☒ Life sciences ☐ Behavioural & social sciences ☐ Ecological, evolutionary & environmental sciences

For a reference copy of the document with all sections, see nature.com/documents/nr-reporting-summary-flat.pdf

Life sciences study design

All studies must disclose on these points even when the disclosure is negative.

Sample size	Sample size was determined by literature research and based on our own previous explorative studies, allowing to achieve sufficient and reliable statistical investigation. Due to the very high intrinsic reproducibility of NMR and our automated ultrasound extraction procedure the state of the art for NMR-based metabolomics allows to have minimum n=3 (for example, a recent study here https://link.springer.com/article/10.1007/s00216-021-03860-0), while we aimed to improve the reliability and reproducibility assessment of our study by using n=4 as minimum.
Data exclusions	Data was excluded only in one condition case, due to a technical sample preparation/acqusition error, leading to insuficient NMR spectroscopy signal readout. This criteria for data exclusion was decided before the study in order to report only technically reliable spectra readout. No other data was excluded from this study.
Replication	The reproducibility of our findings is confirmed. The findings are consisted with our previous work where the same method was applied on different set of samples and conditions: https://www.thno.org/v11p0470.
Randomization	Randomization was not relevant, since we used mice from the same genetic background, same age, same sex and same treatment/model for the current study.
Blinding	Blinding was not relevant to our study due to specific set out research question and well-established experimental protocol.

Reporting for specific materials, systems and methods

We require information from authors about some types of materials, experimental systems and methods used in many studies. Here, indicate whether each material, system or method listed is relevant to your study. If you are not sure if a list item applies to your research, read the appropriate section before selecting a response.

Materials & experimental systems

n/a	Involved in the study	
☐	☒	Antibodies
☒	☐	Eukaryotic cell lines
☒	☐	Palaeontology and archaeology
☐	☒	Animals and other organisms
☒	☐	Clinical data
☐	☐	Dual use research of concern

Methods

n/a	Involved in the study	
☒	☐	ChIP-seq
☒	☐	Flow cytometry
☒	☐	MRI-based neuroimaging

Antibodies

Antibodies used	Cluster of differentiation 3 protein complex (CD3, Clone SP7, DCS Innovative Diagnostik-Systeme GmbH u. Co. KG, Hamburg, Germany).<br>Myeloperoxidase (MPO, Anti-Myeoloperoxidase Ab-1, Lab Vision UK, Ltd., Newmarket, Suffolk).<br>Ionised calcium-binding adapter molecule 1 (Iba1, Abcam, Cambridge, UK).
Validation	The specificity of the reaction was investigated using positive and negative controls during the immunohistochemistry reaction. Spleen was used as positive control for CD3, MPO and Iba1. Additionally, we used a robust automatic immunohistochemistry system for immunohistochemistry staining.

Animals and other research organisms

Policy information about studies involving animals; ARRIVE guidelines recommended for reporting animal research, and Sex and Gender in Research

Laboratory animals	C57BL/6J mice, Jackson Laboratory (Bar Harbor, USA), 8 weeks old.
Wild animals	This study did not involve wild animals.
Reporting on sex	Studies have shown that sex plays a significant role in immune response and metabolism (1). In order to keep the variance between the experimental animals as low as possible, only female mice were used for this study. This reduces the scattering of the data and enables comparability with the previous studies. The choice of the female mice is also based on the fact that male mice often fight for territory after sexual maturity, resulting in scratching and biting marks on the skin. This would affect greatly the model of cutaneous hypersensitivity reaction used in this study.<br>(1) Klein, S.L. & Flanagan, K.L. Sex differences in immune responses. Nat Rev Immunol 16, 626-638 (2016).
Field-collected samples	Our study did not involve samples collected from the field.
Ethics oversight	All experimental protocols were approved by the Regierungspräsidium of Tübingen.

Note that full information on the approval of the study protocol must also be provided in the manuscript.

Dual use research of concern

Policy information about dual use research of concern

Hazards

Could the accidental, deliberate or reckless misuse of agents or technologies generated in the work, or the application of information presented in the manuscript, pose a threat to:

No	Yes	
☒	☐	Public health
☒	☐	National security
☒	☐	Crops and/or livestock
☒	☐	Ecosystems
☒	☐	Any other significant area

Experiments of concern

Does the work involve any of these experiments of concern:

No	Yes	
☒	☐	Demonstrate how to render a vaccine ineffective
☒	☐	Confer resistance to therapeutically useful antibiotics or antiviral agents
☒	☐	Enhance the virulence of a pathogen or render a nonpathogen virulent
☒	☐	Increase transmissibility of a pathogen
☒	☐	Alter the host range of a pathogen
☒	☐	Enable evasion of diagnostic/detection modalities
☒	☐	Enable the weaponization of a biological agent or toxin
☒	☐	Any other potentially harmful combination of experiments and agents
